# Dot-ring nanostructure: Rigorous analysis of many-electron effects

**DOI:** 10.1038/srep29887

**Published:** 2016-07-19

**Authors:** Andrzej Biborski, Andrzej P. Kądzielawa, Anna Gorczyca-Goraj, Elżbieta Zipper, Maciej M. Maśka, Józef Spałek

**Affiliations:** 1 Akademickie Centrum Materiałów i Nanotechnologii, AGH Akademia Górniczo-Hutnicza, Al. Mickiewicza 30, PL-30-059 Kraków, Poland; 2 Instytut Fizyki im. Mariana Smoluchowskiego, Uniwersytet Jagielloński, ul. Łojasiewicza 11, PL-30-348 Kraków, Poland; 3 Instytut Fizyki, Uniwersytet Śląski, ul. Uniwersytecka 4, PL-40-007 Katowice, Poland

## Abstract

We discuss the quantum dot-ring nanostructure (DRN) as canonical example of a nanosystem, for which the interelectronic interactions can be evaluated exactly. The system has been selected due to its tunability, i.e., its electron wave functions can be modified much easier than in, e.g., quantum dots. We determine many-particle states for *N*_*e*_ = 2 and 3 electrons and calculate the 3- and 4-state interaction parameters, and discuss their importance. For that purpose, we combine the first- and second-quantization schemes and hence are able to single out the component single-particle contributions to the resultant many-particle state. The method provides both the ground- and the first-excited-state energies, as the exact diagonalization of the many-particle Hamiltonian is carried out. DRN provides one of the few examples for which one can determine theoretically all interaction microscopic parameters to a high accuracy. Thus the evolution of the single-particle vs. many-particle contributions to each state and its energy can be determined and tested with the increasing system size. In this manner, we contribute to the wave-function engineering with the interactions included for those few-electron systems.

Few-electron systems represent a very interesting topic in quantum nanophysics^1^, as their studies are at the forefront of nanoelectronic applications^2^, e.g., as single-electron transistors^3^,^4^ or other devices^5^,^6^,^7^. Recently, the basic issue of the wave-function manipulation has been raised on the example of quantum-dot—ring nanostructure, DRN^8^ (cf. Fig. 1). DRN is composed of a quantum dot (QD) separated from a surrounding concentric quantum ring (QR) by a tunneling barrier *V*_0_. This structure has been chosen because of tunability of its properties. When compared to simpler systems, such as quantum dots or quantum rings, DRN offers exceptionally high degree of control of the spatial distribution of the electron wave functions. There are several ways to modify the distribution of the wave functions by changing the shape of the confining potential and the selected method depends on the particular realization of DRN. Nowadays technology enables a precise control of this potential, both at the fabrication stage as well as dynamically while operating the device. The confining potential can be defined with the help of many different techniques. It has already been produced via droplet epitaxy^9^,^10^,^11^. The growth procedure is based on the pulsed irradiation of group-V element to group-III element nanoscale droplets for transforming them into various nanostructures with the desired shape, size and dimensionality. Another possibility is to fabricate a core-shell nanowire^12^ with a potential barrier between the core and the shell. DRN can then be produced by cutting a slice of the nanowire. DRN can also be produced electrostatically, e.g., within a two-dimensional electron gas by placing two planar concentric gates, a circular one surrounded by a second ring-shaped, on the top of it^8^,^13^,^14^,^15^. One-electron properties of DRN depend mostly on the relation between the confining potential of QD and QR parts^8^,^16^. If the QD potential is significantly deeper than the QR potential, the ground state electron wave function is located in the QD part. Then, its size is much smaller than the size of the DRN. If one attaches leads to DRN, tunneling rate between the DRN and the leads will be very small. On the contrary, if the QR potential is relatively deeper, the ground state wave function is mostly in the peripheral part of the DRN with a strong coupling to the leads. In this manner by changing the relative position of the QD and QR potentials one can easily control the transport properties of DRN^17^. Modifications of the QD and QR potential allow one to control also, e.g., orbital and spin relaxation times^8^. If the QD potential is sufficiently deep, both the ground and the first excited states are located in the QD. The matrix element between these two states is large, what leads to a fast relaxation. For a smaller difference between positions of the QD and QR potentials, the ground state remains in the QD, but the first excited state location moves over to the QR region. The matrix element between the ground and excited states decreases significantly and hence the relaxation rate drops a few orders of magnitude^8^,^13^. With a further increase of the QD potential also the ground state location moves over to the QR and the overlap of the states increases again and so does the relaxation rate. In refs 8, 18 and 19 it has been shown that this allows to control also other DRN properties such as the optical absorption or spin and orbital relaxation rates. All the mentioned above possibilities to control DRN’s properties have so far been demonstrated only for a single electron occupying the structure. In this context, an interesting question arises as to what happens if the multi-electron states are involved (e.g., with the number of particles 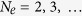).

The standard approach that allows one to take into account Coulomb correlations in nanosystem is the so-called *constant-interaction* (CI) *model*^1^. In this approach it is assumed that all the Coulomb interactions of an electron in a nanosystem with all other electrons can be parametrized by a constant capacitance *C*. Another assumption is that the single-electron energy spectrum is not changed by the presence of the interactions. In many cases, these simplifications work quite well. However, in contradistinction to, e.g., quantum dots, the single-electron states in DRN may have very different shapes (e.g., their maxima can be in either QD or QR). Moreover, small changes of the confining potential can significantly affect the wave functions, e.g., moving them between QR and QD parts of DRN. In the following we demonstrate that the wave functions are also sensitive to interparticle interactions. Therefore, the approximations used in the CI model cannot be valid in the case of DRN and more precise methods have to be used. Such problem has been addressed earlier^15^, where the spin and the charge switching in the applied magnetic field have been analyzed in detail. The results demonstrate that such model system can reflect the situation encountered in experimentally constructed devices of DRN type^9^,^10^,^11^.

In this paper our aim is somewhat more fundamental. Namely, we include in a rigorous manner the interelectronic interactions for a preselected (finite) basis of single-particle states, appropriate for the system geometry. The experimentally controlled parameter is the gate electrostatic potential *V*_QD_ of the quantum dot relative to that of the ring. We determine next the system energy for *N*_*e*_ = 2 and 3 electrons, as well as the many-particle wave function. This, in turn, allows us to construct the particle-density profiles and in particular, the partial contribution of the component single-particle-state products to the many-particle ground- and the first-exited-states. Such a decomposition into the single-particle product components is possible in the method we use, in which we combine the first- and second-quantization schemes of determining the many-particle state. In essence, the many-particle Hamiltonian in the occupation number representation (Fock space) is diagonalized starting from the preselected set of single-particle states in the Hilbert space providing the scenario for possible multiple-particle occupation configurations. For the original presentation and application of the method to various nanoscopic systems see^20^,^21^,^22^,^23^. Explicitly, we predetermine the lowest 10 single-particle states 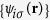 for given shape of DRN potential. Those single-particle states (obtained numerically for given topology of the device) are used as an input to define the field operators (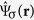 and its Hermitian conjugate counterpart 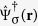, respectively) by the prescription


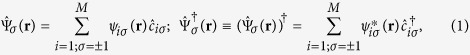


where 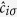 (and 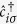) are the annihilation (creation) operators of particle in the single-particle state 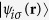. Note that the number *M* of states included in definition of the field operator is selected in such a manner that any further enrichment of the single-particle basis 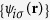 does not change quantitatively the characteristics of the ground and the first excited states. Here, it is sufficient to take *M* = 10. In effect, no problem connected with the basis incompleteness should arise. This formal point will also be discussed *a posteriori*.

The next step is to define many-particle Hamiltonian in the second-quantization language in a standard manner (cf. e.g. ref. 24) which we diagonalize in a rigorous manner. This last step allows for determination of the system global characteristics such as the total system energy, the multiparticle wave function, the particle density profile *n*(**r**), the total spin, and the energies of the transition between the states, e.g., the spin singlet—triplet transition for *N*_*e*_ = 2, etc. What is equally important, we calculate **all** the microscopic interaction parameters *V*_*ijkl*_, including the 3— (e.g., *V*_*ijki*_) and 4-state parameters *V*_*ijkl*_, i.e., those with all the indices different. In result, we can discuss explicitly the importance of those nontrivial terms, which are often neglected even in many-particle considerations^21^,^22^,^23^. We believe that this last result, coming from our method should be taken into consideration, as those interactions are often non-negligible, to say the least. In any case, they should be evaluated to see their relevance, at least in model situations.

The structure of the paper is as follows. We define first the Hamiltonian and detail the method of calculations. Next, we discuss the basic characteristics of the multiparticle states, as well as determine the values of all nontrivial microscopic parameters. Finally, we determine the energy of the singlet-triplet transition (for *N*_*e*_ = 2), as well as discuss the doublet-quadruplet transition for *N*_*e*_ = 3, which should be detectable in the microwave domain. At the end, we discuss briefly the application of our results to determine the optical transitions and, e.g., the transport of electrons throughout such system. In Supplementary Material we display the shapes of the starting single-particle wave functions, provide detailed numerical values of the 3- and 4-state interaction parameters, as well as display the detailed system characteristics for selected values of *V*_QD_. In particular, in Supplement D we show the first two states degeneracy which contains a chiral factor to it, depending on the number of ways the orbital currents can be arranged for given conserved total quantum numbers 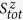, 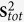 and 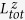.

## Problem and Method

We start from the single—particle solution of the Schrödinger equation for the DRN system parametrized as in^17^. Therefore, the set of the single-particle eigenfunctions 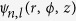 in the cylindrical coordinates, being the solution for the one-electron DRN picture, is assumed at the start^8^,^17^,^19^. The many—particle problem in which electrons are described by the second quantizied Hamiltonian has the standard form^24^.


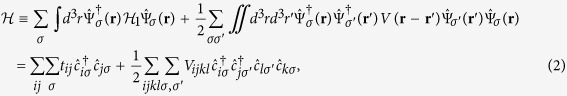


where 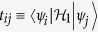 and 
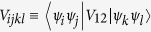
 are the microscopic parameters which are calculated in the basis 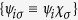. The single-electron Hamiltonian 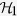 is given by


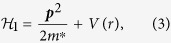


where *V*(*r*) is the confining potential of DRN. The spin—orbit interaction is neglected. In effect, the changes with respect to the corresponding one-particle considerations^8^,^17^ are induced solely by the interparticle interactions. The symbols 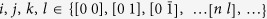 represent quantum number pairs referring to a single—particle solution [*n l*]^17^. One specific feature of the problem should be noted. Namely, since the single-particle wave-functions 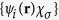 represent the eigenfunctions of the single-particle Hamiltonian, i.e., 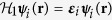, the first term in (2) is explicitly diagonal, i.e., 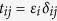. Therefore, the diagonalization of the Hamiltonian (2) means that such a procedure is applied to the interaction part (the second term).

To solve many-electron problem for a fixed number *N*_*e*_ of electrons, one must proceed in two steps:

1. Compute explicitly one- and two-body microscopic parameters, 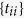 and 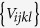, respectively.
2. Diagonalize the Hamiltonian (2) in the Fock space.

Each of these steps is discussed below. But first, we have to define the starting single-particle wave functions in the real-number domain.

### Change to the real single-particle basis functions

Eigenfunctions 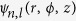
—by their nature—form an orthogonal and normalized single-particle basis of planar rotational symmetry^8^,^17^,


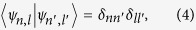


where in the cylindrical coordination system 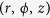 we have that


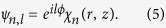


As the microscopic parameters are to be calculated numerically (since the explicit analytical form of the single-particle wave functions is not known), it is convenient to deal with the real-space basis. Hence, we utilize the real representation, exploiting in fact the cylindrical geometry of problem, namely


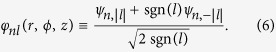


### Microscopic parameters computation

The transformation (6) preserves both the orthogonality and the normalization of starting wave functions and can be applied to the computation of the microscopic parameters defining Hamiltonian (2). Evaluation of single—particle parameters *t*_*ij*_ is performed in terms of integration in the new basis, namely


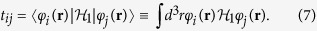


However, as said above, since eigenproblem of one electron is solved^17^, the eigenvalues *t*_*ii*_ = *ε*_*i*_ are known (cf. Fig. 2). Furthermore, the elements *t*_*ij*_ for 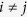 vanish also after the basis transformation to the form (6). For the sake of clarity, we define 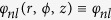 and label 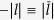 to write explicitly that


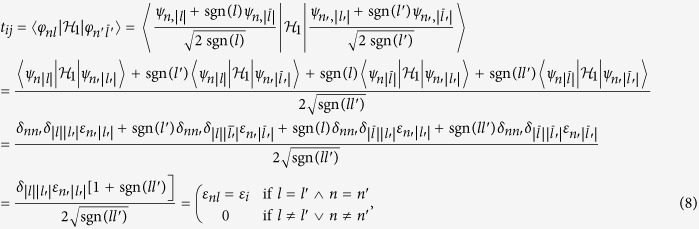


where 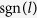 is the sign function. We also utilize symmetry of the single-particle solution, i.e., 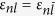.

Now, the two-body (four-state) integrals *V*_*ijkl*_ are expressed as


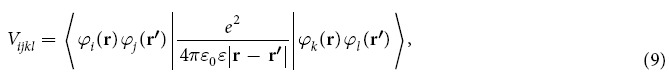


where *e* is the electron charge, *ε*_0_ is the vacuum dielectric constant and *ε* = 12.9 is the relative dielectric constant, taken here for the *GaAs* system. Their explicit determination is required for a further Hamiltonian matrix construction. Here, we use the Coulomb potential 1/*r* screened only by the medium with relative dielectric constant *ε*. This assumption is valid when DRN is formed, e.g., by droplet epitaxy or by slicing a core-shell nanowire. However, for an electrostatically defined DRN the presence of image charges in the electrodes would screen further the interaction. The same effect would be observed if a central gate is used to control the shape of the confining potential by changing *V*_*QD*_. Since screening is more important at large distances, we expect that it will decrease the energy of states with electrons located in the QR part of DRN. The details, however, would depend on the screening radius that, in turn, depends on the particular geometry and method of fabrication of DRN. Therefore, in the following we neglect this effect, as we are interested in providing generic properties of the system.

Up to four-state integrals in Eq. (9) involve six-dimensional integrals and therefore, standard numerical integration techniques are not suitable for this task. Instead, the Monte-Carlo integration scheme has been applied. For that aim, we use CUBA library^25^, selecting the *suave* algorithm for the integrals calculations. The procedure is standard and the accuracy of such integration is typically 0.005 meV or even better.

### Method: diagonalization of the multiparticle Hamiltonian

We start from the occupation number representation of the multiparticle states in the Fock space in the following form


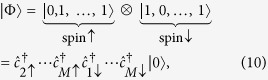


where *M* is the number of states. We find explicitly all the possible states for *N*_*e*_ electrons and thus are able to build up Hamiltonian matrix out of (2) by calculating all the averages 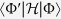. We diagonalize the resultant matrix using the *QR decomposition* of the Gnu Scientific Library (GSL)^26^. The usage of Lanczos algorithm is not efficient in this case, as both the ground and the first excited states can be highly degenerate. *The QR decomposition*, as well as the GSL library, operate with relatively small matrices (of dimension not exceeding 10^5^ × 10^5^ elements), but this is not the number of states to be reached for small number of electrons, even for a relatively large number of sinle—particle wave—functions included in the starting basis (1).

For the purpose of these calculations we employ also our library *the Quantum Metallization Tools* (QMT)^27^, proved to be efficient for similar problems^22^. Explicitly, the calculations of the parameters *t*_*ij*_ and *V*_*ijkl*_ in (2) have been carried out with the help of the Monte-Carlo (MC) integration method described in^25^. The accuracy of their evaluation is estimated as 0.005 meV. The validity of application of MC in the current context was tested by means of a numerical computation of the *on*—*site* 1*s* electron—electron interaction for the Slater function, for which an analytical formula exists.

## Results: Two- and Three-Electron States

### Basic characteristics

We are interested in calculating the system observables. In this Section we present the results for basic quantities, in this case the energy, and the total electronic density 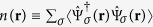 in the many-particle state. The states are characterized by the conserved quantities, i.e., the *z*-component *L*^*z*^ of the angular momentum, the total spin 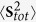, and its *z*-component 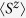. Explicitly, in Fig. 3 we plot the ground and excited state energies for *N*_*e*_ = 1, 2, and 3 (curves from bottom to top, respectively). The energy increases substantially with each particle added to the system, as expected for the Coulomb system of charges. The single-particle part of the potential energy 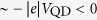 represents a substantial contribution for its value ~few *eV*_QD_, comparable to that introduced by the repulsive interaction for *N*_*e*_ = 2 and 3. Before entering into a detail microscopic analysis, we would like to relate first our results to a quasiclassical approach. Namely, if the QD potential is sufficiently deep, many electron states are localized in the central part of the DRN and the entire system effectively looks like a small multi-electron QD. It turns out that in this case the CI model can reproduce the actual energy spectrum. According to this model, the ground-state energy for two electrons is given by 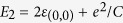, where 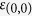 is the (two-fold degenerated) single-electron ground-state energy and 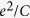 is the interaction energy. Taking the relevant values for 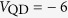 meV from Fig. 3 one can calculate 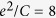 meV and the capacitance C ≈ 20 aF. This value corresponds to the self-capacitance 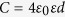, with *ε* = 12.9 for GaAs, of a free disc with diameter 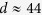 nm, almost exactly the diameter of the flat bottom of the potential of the QD part of the DRN (cf. Fig. 2). For 3 electrons the CI model provides the energy of the ground state *E*_3_ higher than *E*_2_ by 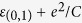, where 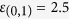 meV is the energy of the first single-electron excited state. This formula gives *E*_3_ = 13.0 meV, very close to the actual ground state energy for *N*_*e*_ = 3 that equals 12.9 meV. However, for higher values of *V*_*QD*_, i.e., when some of single-electron wave functions are moved over to the QR part of the DRN, this simple model is not accurate enough. For example, for *V*_QD_ = 6 meV the energy of the ground state for two electrons equals 13.1 meV, what gives the capacity *C* ≈ 120 aF. Then, the CI model predicts the ground state energy for *N*_*e*_ = 3 equal to 20.5 meV, whereas the actual value is *E*_3_ = 22.3 meV.

### Two electrons

Here we present electronic density, as well as 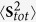 and 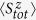 for the ground and first excited states of DRN for 2 electrons. The ground state is always the spin-singlet *S* = 0 (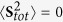) state, whereas the first excited state is the spin-triplet *S* = 1 (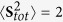).

As can be seen in Fig. 4, with the increasing *V*_QD_ from −4 meV to +6 meV there is a gradual shift of dominant part of the electron density from QD to QR. If the bottom of the central part of the confining potential is very low, the electron density is the largest within the dot part of DRN as attractive *V*_*QD*_ in this case is comparable or larger than the interaction energy. In this regime [row 1) in Fig. 4] the single particle state with *n* = 0 and *l* = 0 gives the main contribution to the two-particle state. When *V*_*QD*_ becomes less negative the Coulomb interaction partially “pushes out” the electron density towards the outer part of the DRN [row 2) in Fig. 4]. It is realized by increasing the contribution of the single particle state with *n* = 1 and *l* = 0 to the two-particle wave function. With further increase of *V*_*QD*_ it becomes energetically favourable to reduce the occupancy of QD, i.e., in the area where the interaction is strong due to a strong confinement in a small area. As a result, the electron density increases in QR and single-particle states with nonzero angular momenta become occupied. Finally, for *V*_*QD*_ = 4 meV only the states in QR are occupied.

A similar evolution can also be observed for the excited states. Fig. 5 shows the first excited state for *V*_QD_ = −6 meV. With increasing value of *V*_QD_ also the excited state is moved over to the ring part of DRN, similarly to the ground state. The evolution is presented in Supplementary Figs S3 and S4.

The contribution of the 2–3 first single-particle functions 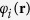 out of *M* = 10 states to *n*(**r**) is usually predominant. Inclusion of e.g., *M* = 18 states in (1) does not change practically the results. This last circumstance means that the interaction involves only a relatively small number of two-particle components 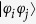 in the resultant two-particle state 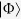, at least for the lowest excited states of the system.

### Three electrons

Next, we present electronic density, as well as the squares of the total spin and the spin component along an arbitrarily selected *z* axis for the ground and the first excited states of DRN for 3 electrons (cf. Figs 6 and 7). The ground state is the state with the total spin *S* = 1/2 (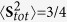) for 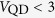 meV or *S* = 3/2 (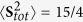) for 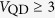 meV. For the high spin state, a redistribution of the density *n*(**r**) into the products of single-particle component is more involved, as one would expect, whereas for *S* = 1/2 the state is composed of the dominant pair-singlet state and the third electron in a higher orbital with the dominant ring contribution.

Parenthetically, it would be interesting to calculate the transport properties via tunneling through the DRN with *N*_e_ = 2 as this would involve cumbersome intermediate state with *N*_e_ = 3. Depending on *V*_QD_, the tunneling probability is allowed (for *S* = 0) and substantially suppressed when *S = *1 (in applied field). Such effects should be analyzed separately as they involve an analysis of electronic transitions between the many-electron states.

### Coulomb-interaction parameters

We now turn to the most basic aspect of our present work. Namely, we calculate all possible microscopic interaction parameters *V*_*ijkl*_ appearing in (2). Those parameters appearing in the microscopic parameters reflect various quantum processes encoded in the starting Coulomb repulsion. This procedure should allow us to determine a coherent and exact many-particle physical picture with concomitant information concerning the importance of various classes of interaction terms, as expressed via the respective one-, two-, three-, and four-state terms. We start by rewriting the starting Hamiltonian (2) to the following form


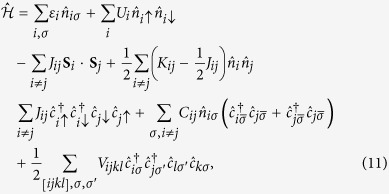


where the first 6 terms represent one- and two-state interactions^20^,^23^, respectively, and 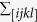 refers to sum over indices with at least three of them being different. The first question relates to the magnitude of the intrasite Hubbard interaction, 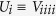 (cf. Fig. 8 and Table S1 in Supplem. Material), the generic term in the Hubbard model, as compared to the inter-state repulsion 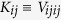 (cf. Fig. 9 and Table S2 in Suppl. Mat.), the exchange energy 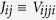 (cf. Fig. 9 and Table S3), and the so-called correlated hopping 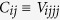 (cf. Fig. 9 and Table S4). In the present situation, the inclusion of three- and four-index interaction parameters 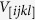 (cf. Fig. 9 and Table S5) is of the crucial importance, as these parameters are usually omitted in the models describing various quantum devices. The reason for including them is due to the circumstance that in a few-electron system there is no screening and thus, in principle, all the terms may become relevant. In any case, on the example of DRN we can see explicitly the role of all consecutive terms, what is, in principle, of fundamental importance for a reliable modeling of the nanodevices. These last terms proved to be nonnegligible as shown in Figs 10 and 11 (cf. also Table S5), and can become even of comparable magnitude to the exchange energy.

Visible in most of the cases in Figs 8 and 9 are the rapid changes of the microscopic parameters which coincide with the single-particle level-crossings observed in the single-particle levels (cf. Fig. 2), but these do not influence in any essential manner the resultant many-particle picture, as may be explicitly seen in Fig. 2, where we observe a smooth evolution with changing *V*_*QD*_.

### Two-state versus the three and four-state interaction contributions

We illustrate next the role of the pairwise vs. 3- and 4-state interactions with their paramters displayed in Figs 8 and 9. For that purpose, we draw in Fig. 10a the exemplary profile of the electron density cross section *n*(*r*_*x*_) for 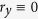, for *N*_*e*_ = 2 without and with the 3- and 4-state interactions included. The role of the latter terms is essential. As expected, with those interactions included, the electrons are pushed to the ring region in that situation. On the contrary, the role of the 3- and 4-state terms is not so crucial when evaluating the ground state energy (cf. Fig. 10b). Therefore, one sees that the 3- and 4-state interactions will be of primary importance when evaluating the matrix elements between the states.

To determine explicitly the role of the three- and four-state interaction terms we have plotted in Fig. 11 the particle density profile with and without inclusion of them. We see that their role is crucial. Note that each of the curves has the same area equal to 2 (the number *N*_*e*_). The apparent inequivalence arises from the circumstance that the ring part encompasses effectively a larger volume (here only a single cross-section *n*(*r*_*x*_) is plotted). So all the interaction terms contribute in a nontrivial manner to the many-particle wave-function engineering! Also, one can compose a resultant many particle state out of the products of the single-particle basis states and the leading terms are


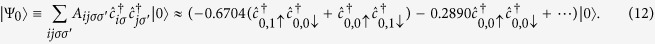


The complete list of the leading coefficients 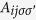 for the ground state spin singlet is provided in Table 1. Note that their values are the same for the components 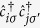 and 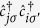, of that singlet state. Essentially, the decomposition (12) with the complete list of the coefficients (cf. Table 1) provides the same type of expansion as that appearing in the Configuration Interaction method^28^. Here, a particular combination of the pair products of the creation operators represents a single Slater determinant of the single-particle wave functions and the respective numerical values of the coefficients describe the weight of each two-particle Slater determinant state. From Table 1 we see that only limited number of such states matter in this (and other) cases. This means that if the number *M* of single-particle states in (1) is selected properly, the obtained results for many-particle states and their eigenvalues can be achieved to a very high accuracy. Here, it has been sufficient to choose *M* = 10 for *N*_*e*_ = 2, 3. For the state (12) this results in having 24 leading coefficients listed in Table 1, i.e., the state can be represented well by 24 component states composing that state. For larger values of *N*_*e*_, the method is also workable, but the value of *M* must be selected with care.

### Electronic transition from the ground to the excited state

To flash on the importance of the system behavior, we examine the possibility of changing the state of electrons in DRN via an intraband photo-excitation for *N*_*e*_ = 2. From the experimental point of view, the possibility of changing the probability of electrons to be in QD or QR is of importance. This can be realized by a microwave radiation absorption, as illustrated in Fig. 12. The selection rules are fulfilled as we are starting from the state 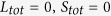 and ending in the state 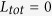, *S*_*tot*_ = 1, where *L*_*tot*_ and *S*_*tot*_ represent the orbital and spin state of the system, respectively.

A detailed analysis of the interstate transition drawn in Fig. 12 may have important principal information about 3- and 4-state interactions. Namely, by studying DRN systems of a variable size, one should see their diminishing role with the increasing system size. Such measurements when performed, can be readily analyzed within the exact solution provided here (the codes for the analysis of DRN for 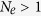 are available at https://bitbucket.org/azja/qmt).

## Scaling of Results with Variable DRN Size

**Figure 1:**
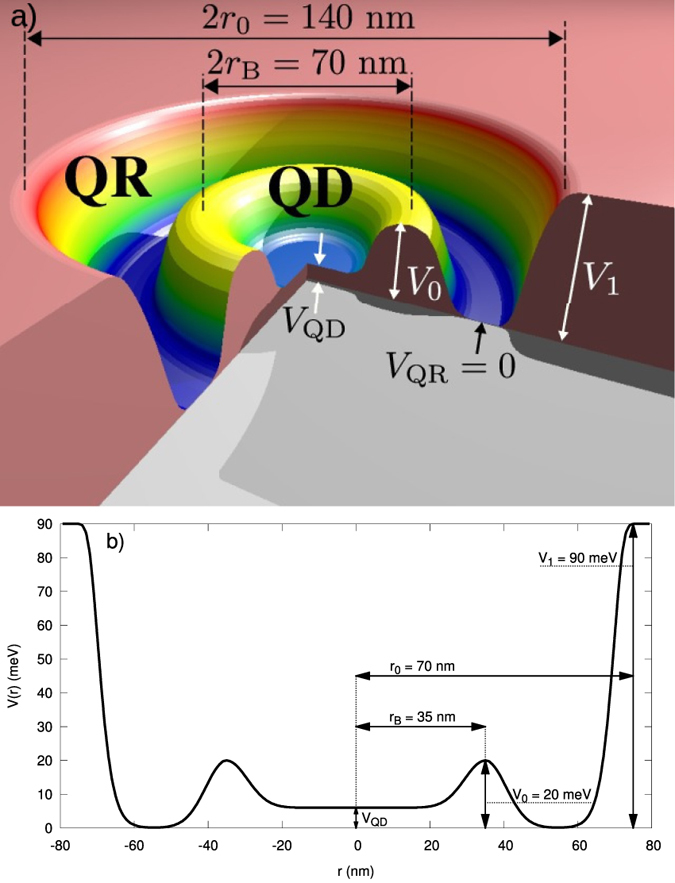
(a) Schematic representation of quantum-dot (QD)—ring (QR) structure into DRN; (b) the shape of the actual single-particle potential energy, with the corresponding values taken in the analysis.

**Figure 2:**
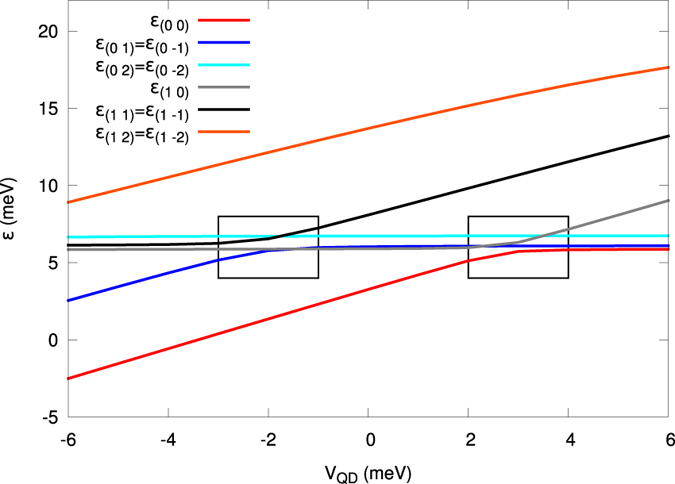
Single-particle energies {*ε*_*i*_} for the first ten wave-functions versus QD potential energy *V*_QD_. One can see that some the energies vary with the value of *V*_QD_, whereas the others are independent of *V*_QD_. States in the former group are located in the QD part of the DRN and the latter states in the QR part. Note the two regions of the level crossing or anticrossing (framed). In these regimes, some of the states, with the increasing *V*_QD_, change over from the QD to the QR as the dominant regions (after^17^).

**Figure 3:**
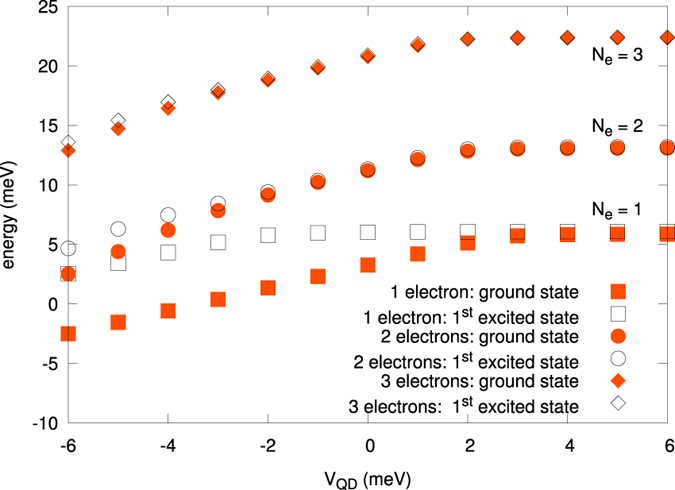
Ground and first excited state energies for *N*_*e*_ = 1, 2 and 3 electrons in DRN versus the QD potential energy. The single-particle energy (bottom squares) is provided for comparison. The interelectronic interactions alter essentially the resultant energies. Note that roughly the energy for *N*_*e*_ = 3 increases with respect to the case *N*_*e*_ = 2 by the factor 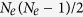.

**Figure 4:**
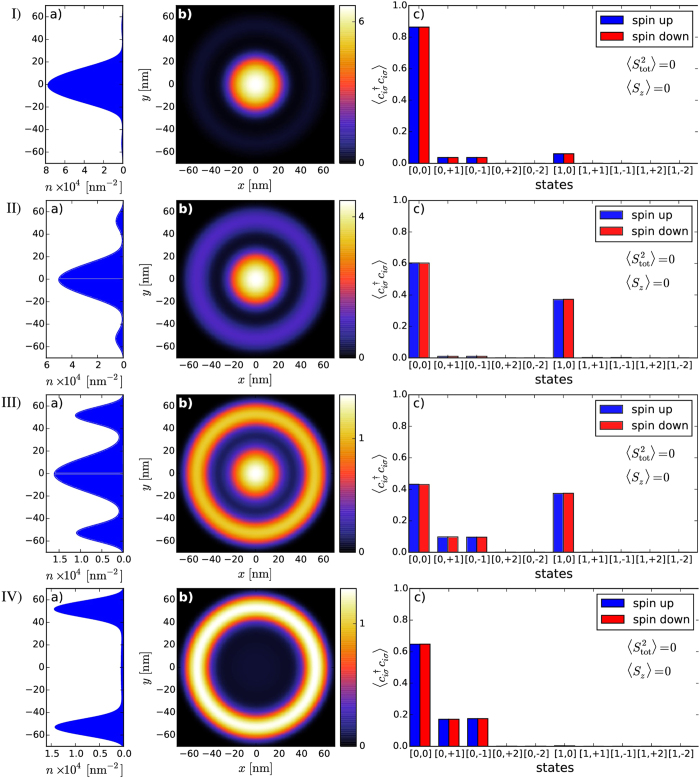
Evolution of the electronic density profiles *n*(*x,y*) [(a) and (b)] and the occupancy 
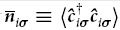
 of the single-particle states c) composing the many particle state for *N*_*e*_ = 2. Rows I–IV correspond to *V*_QD_ equal to −4 meV, −2 meV, 2 meV, and 4 meV, respectively. First ten single-particle states have been taken into account to compose the resultant two-particle state for given *V*_QD_. The occupancy of the higher in energy single-particle states is negligible; those are shown in c) for completeness.

**Figure 5:**
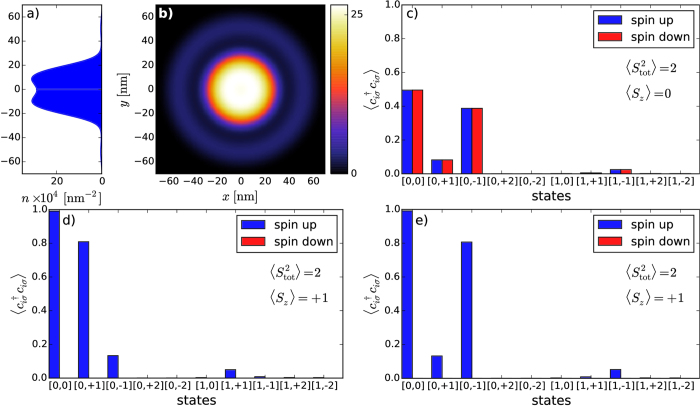
The same as in Fig. 4 for *N*_*e*_ = 2, but for the first excited state with *V*_*QD*_ = −6 meV. This state is six-fold degenerate and the presented electron density is averaged over all the six states. In panels (c) to (e) the occupancy of single-particle states is shown for half of the states in the basis. For the state with 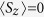 [(c)] there exists a counterpart with exchanged single-particle contributions [0, + 1] and [0,−1]; for the states with 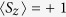 [(**d**) and (**e**)] their counterparts with 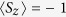 have the same contribution.

**Figure 6:**
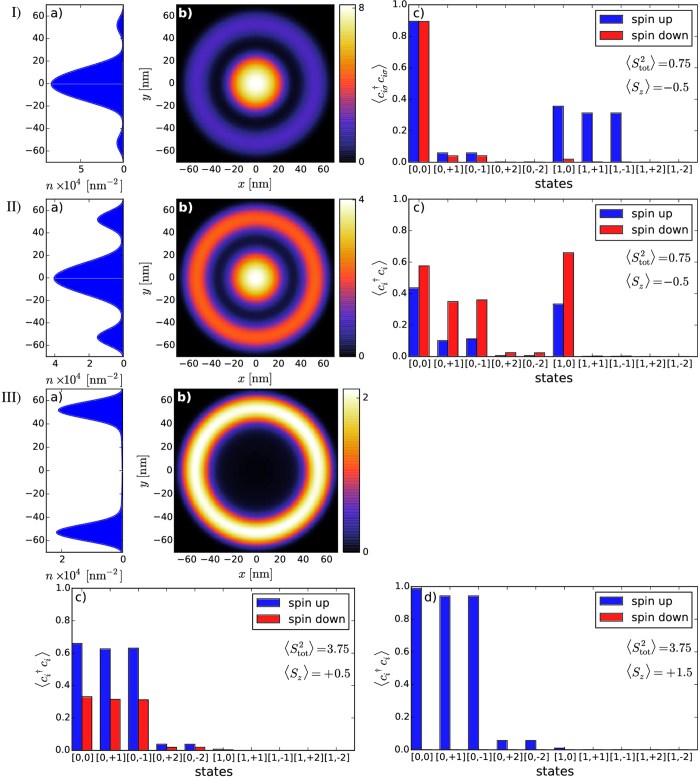
Electronic density profile *n*(*x,y*) [(a), (b)] and occupancy 
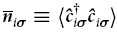
 of the single-particle states included in the calculations [(c), d)] for the ground state for *N*_*e*_ = 3. Rows I–III correspond to *V*_QD_ equal to −6 meV, 1 meV and 4 meV, respectively. For *V*_QD_ = −6 meV the total spin is 1/2. Two electrons are forming a singlet and located mainly in the dot part, whereas the third (unpaired) electron is located further away, as seen by the presence of the spin-polarized subsidiary occupancy maxima in 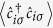 (cf. c). This state is degenerate, its counterpart has exchanged the occupancy of spin-up and spin-down states.

**Figure 7:**
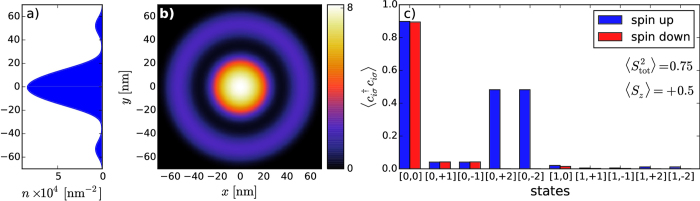
The same as in Fig. 6 for *N*_*e*_ = 3, but for the first excited state. The electronic density is almost exactly the same, but the occupancy 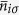 of the single-particle states is different. The state eigenenergy is 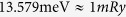.

**Figure 8:**
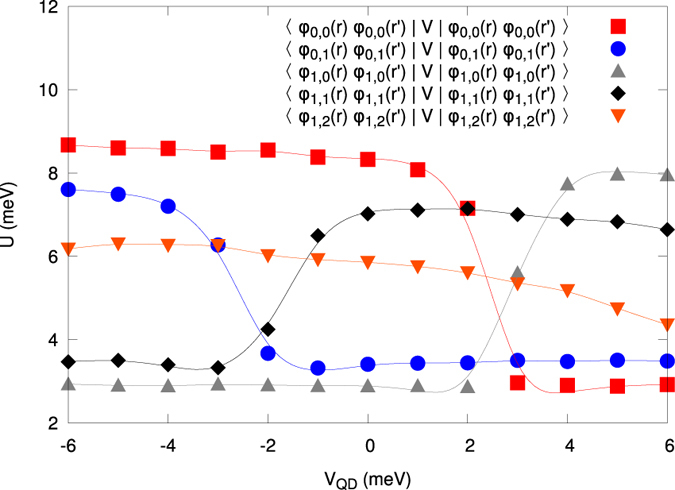
Values of the Hubbard (intrasite) repulsion 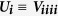 vs the tuning parameter *V*_QD_ for different states, as marked. These values are in some cases comparable to the single-particle energy, so the interelectronic correlations are very important then. The continuous lines are guide to the eye to visualize the tendency of the calculated points. The nonmonotonic behavior is due to the level crossing depicted in Fig. 2.

**Figure 9:**
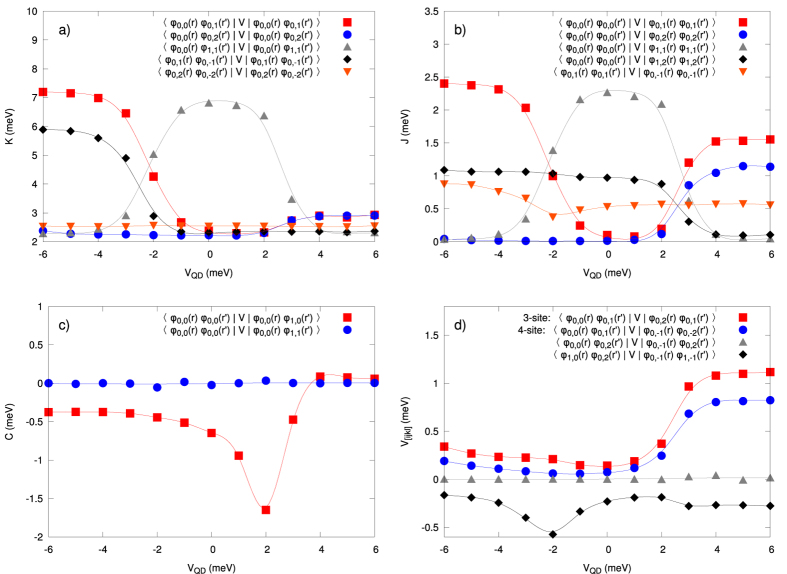
(**a)** Two-state (interstate) Coulomb repulsion amplitudes 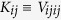 vs *V*_QD_. A rapid change of their values can be related to the left level (anti)crossing specified in Fig. 2 and the concomitant change of the single-particle wave-function symmetry of the corresponding states. Note also that for *V*_QD_ 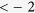 meV some of the values of *K*_*ij*_ can even become comparable to those of *U*_*i*_. (**b)** Exchange integral 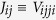 vs QD potential. The convention is that 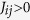 denotes the ferromagnetic spin-spin exchange interaction. **c)** Selected correlated hopping amplitudes 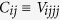 vs QD potential. The terms containing this parameter (the sixth term in (11)) lead to the nonorthogonality of the starting single-particle basis 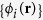, i.e., to the hopping between those states with a double occupancy in either initial of final states. (**d)** Selected three- and four-site *V*_[*ijkl*]_ parameters vs QD potential. They become comparable to *J*_*ij*_ for 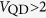 meV, where the second level crossing appears, particularly when one takes into account the circumstance that the number of, e.g., four-state terms is relatively large. The continuous lines are guide to the eye.

**Figure 10:**
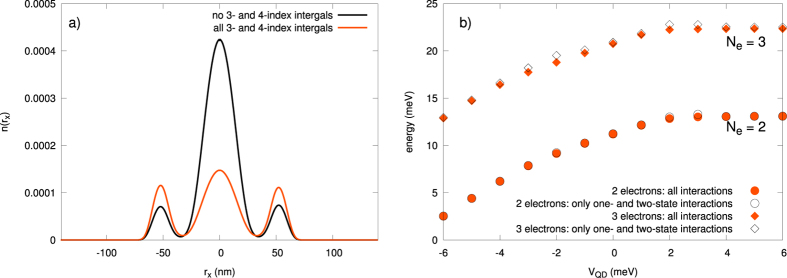
(**a)** Electronic density along the *x* axis (for 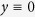) for the case of *N*_*e*_ = 2 and *V*_QD_ = 2 meV, with no 3- and 4-indices integrals included (black), as compared to that coming from our present approach which includes all the microscopic parameters (orange). In the former case, the density is reduced essentially to the dot region. (**b)** The ground state energies for *N*_*e*_ = 2, and 3 without and with the 3- and 4-state interactions included. The role of the latter is of secondary importance for these quantities.

**Figure 11:**
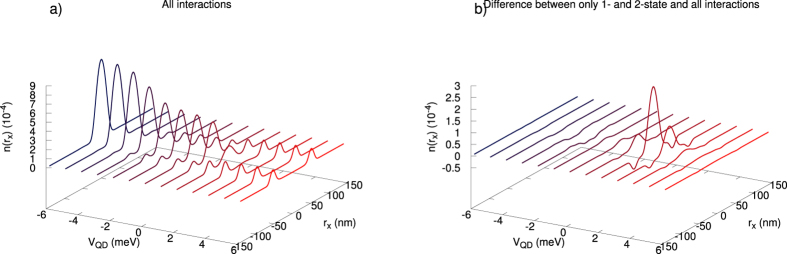
(**a)** Electronic density along the *x*-axis, i.e., on the 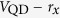 plane (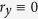), with all the interactions included. (**b)** Difference in the density between the situation with the pairwise interactions taken only and that with all the interaction terms taken into account. The difference is the largest in the *QD* region and for *V*_QD_ at and around zero.

**Figure 12:**
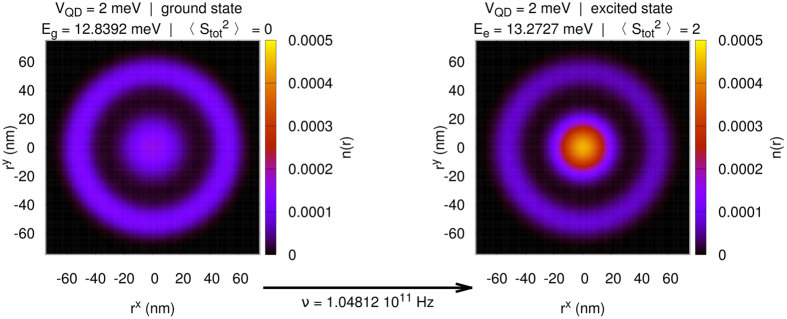
Change in the overall electronic density for two electrons in DRN, for *V*_QD_ = 2 meV, after absorption of a photon of frequency ν = 1.05 · 10^11^ Hz. Note that this excitation is allowed as the change of respective angular orbital and spin momenta are Δ*L*_*tot*_ = 0 and ΔS_*tot*_ = 1.

The results discussed above have been obtained for the DRN size specified in Fig. 1. This size can be regarded as typical for this type of devices^29^. This is because the effective Bohr radius for this material is 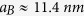 (for GaAs, for which *ε* = 12.9, and 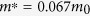), so the DRN size is about 10 times larger and the quantum-dot size is 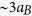. Such size of the dot leads to the circumstance that a substantial number (>10) of hydrogenic-like bound states appear already for this size, which in turn form a rich enough starting basis for defining the field operator (i.e., a reliable basis for many-particle considerations).

**Figure 13:**
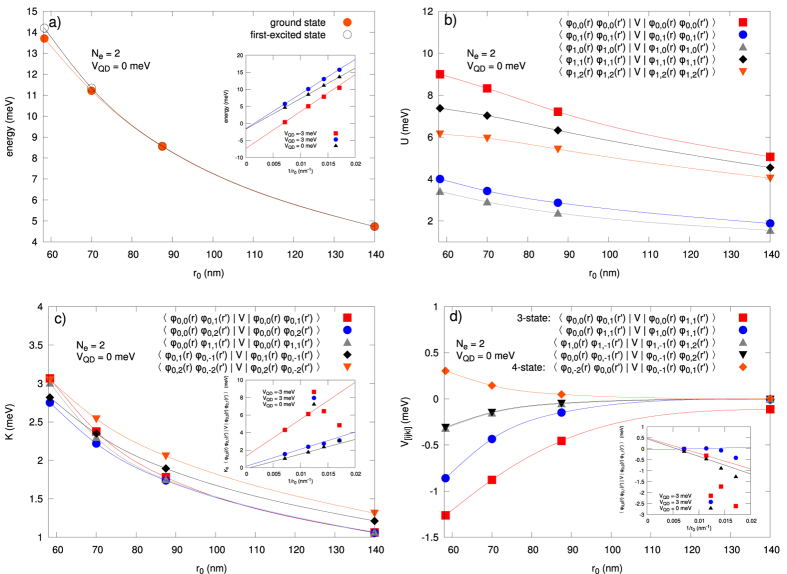
Basic characteristics of DRN with variable size *r*_0_, for *V*_QD_ = 0 and *N*_*e*_ = 2. In the insets: the same characteristics vs 1/*r*_0_ (then also 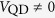 is included). (**a)** ground-state and first excited states energies. (**b)** intrastate interaction *U*. (**c)** interstate interaction *K*. (**d)** selected 3- and 4-state interactions. Magnitudes of all quantities decrease with the increasing *r*_0_.

A natural question may arise to what extent the results are generic, i.e., only weakly quantitatively dependent on the DRN size. To check this we have scaled the results with the varying DRN size *r*_0_. In the panel composing Fig. 13(a–d) we have drawn the principal characteristics as a function of *r*_0_ (in the bulk of the paper *r*_0_ = 70 nm, cf, Fig. 1). In Fig. 13a the exemplary ground-state and the first excited-state energies (for *V*_QD_ = 0 and *N*_e_ = 2) vs *r*_0_ are displayed. With increasing *r*_0_ the interaction energy decreases, so that in the 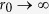 limit the total energy becomes just the sum of single-particle energies. This is illustrated in the inset to this figure, where we have shown that the final-size-type of scaling ~1/*r*_0_ asymptotically reaches zero for *V*_QD_ = 0 and 3 meV, i.e., when the wave function has a significant ring component, and about −6 meV for *V*_QD_ = −3 meV, i.e., when the wave functions are located mostly in the dot part. The energy value varies essentially. In Fig. 13b the values of intrastate interaction *U* for *V*_QD_ = 0 are shown for selected states and decrease approximately linearly with the increasing system size. In Fig. 13c the corresponding interstate values are shown, together with the 1/*r*_0_ scaling in the inset. Again, only the data for *V*_QD_ = 0 and 3 meV scale to zero with 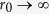, as then the ring size increases. Finally, in Fig. 13d the specified 3- and 4-state interactions are shown, and the same type of conclusion for the scaling 1/*r*_0_ can be drawn for *V*_QD_ = 3 meV. In general, the interaction strength decreases with the increasing size *r*_0_, though the finite-size scaling ~1/*r*_0_ is only observed in special cases. Nevertheless, since the quantities vary in a systematic manner with changing system size, the experimental realization of DRN seems to be feasible and thus the determination of interaction effects possible.

## Outlook

In this paper we have addressed in a rigorous manner the question of importance of the interelectronic interactions/correlations in nanodevices (on example of DRN). The cases tackled explicitly were those with *N*_*e*_ = 2 and *N_e_* = 3 electrons. We have calculated all relevant interaction parameters and their evolution with the tuning parameter, which in this case is the relative potential *V*_QD_ of the quantum dot (QD) with respect to that for the ring (taken as zero). We have proved explicitly that practically all relevant interaction terms are important, as they change essentially the shape of the multiparticle wave function. The situation depends on the size of DRN system. Such feature could be tested experimentally.

To test further the role of many-particle interactions, one can follow the two principal directions. First, the determination of the states in an applied magnetic field and in this manner see the evolution/crossing of many-particle states as the field increases. This topic can become quite interesting as the transition between low and high spin states may turn out then to be quite nontrivial. Second, the charge transport/tunneling processes through DRN can be nontrivial as they should also be connected with the total spin values change when applied field/*V*_QD_ are altered. It has already been demonstrated that in the single electron regime the system can be applied as a switching device (transistor)^17^. Taking into account the possibility of controlling many-particle states, such situation would allow for manipulating the spin-dependent coupling between the DRN and the leads. This, in turn, opens a new area of applications, also in single spintronics, e.g., as spin valves or spin filters. We should see a progress along theses lines soon.

Finally, as mentioned above, one could also vary the system size and see the evolution of the relative roles of single-particle vs. many-particle contributions to the total energy. In a smaller nanosystem electrons will be confined more tightly so that the distances between them will be smaller. It will result in stronger Coulomb interactions. In the simple CI model the interaction energy is inversely proportional to the capacitance of the system, so it would decrease like 1/*d* with the increasing diameter *d*. On the other hand, if we approximate the nanostructure by a infinite circular quantum well, the single-particle eigenenergies are proportional to 1/*d*^2^. As a result, in this naïve picture the role of the single-paricle energies would diminish with respect to the interaction energy when the size of the DRN increases. However, since both the above assumptions, i.e., the CI model, and the infinite circular quantum well, may not be valid for a complex system like DRN, we have explicitly calculated how the one-particle spectrum and the interaction energy change with the DRN’s size, as shown in the panel composing Fig. 13. In this manner, the DRN system may be useful for not only single-electron, but also for many-particle wave-function engineering and associated with it total-spin value changes.

## Additional Information

**How to cite this article**: Biborski, A. *et al*. Dot-ring nanostructure: Rigorous analysis of many-electron effects. *Sci. Rep.* **6**, 29887; doi: 10.1038/srep29887 (2016).

## Supplementary Material

Supplementary Information

## Figures and Tables

**Table 1:** Values of the leading coefficients 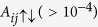 for the case *V*_QD_ = 0 meV, and for the two-particle state (12) for different pairs (*i*, *j*) of states composing this state.

	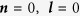	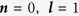	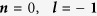	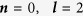	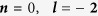	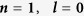	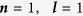	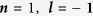	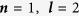	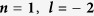
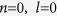	−0.2890		−0.0005			−0.6704		−0.0002		
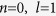		0.0652					−0.0450			
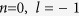	−0.0005		0.0653					−0.0451		
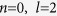				0.0071					−0.0049	
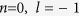					0.0071					−0.0049
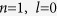	−0.6704					−0.0149				
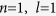		−0.0450					0.0169			
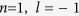	−0.0002		−0.0451					0.0169		
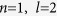				−0.0049					0.0044	
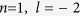					−0.0049					0.0045

## References

[b1] Kouwenhoven L. P., Austing D. G. & Tarucha S. Few-electron quantum dots. Rep. Prog. Phys. 64, 701, 10.1088/0034-4885/64/6/201 (2001).

[b2] Ihn T. Semiconductor Nanostructures, 10.1093/acprof:oso/9780199534425.001.0001 (Oxford University Press, 2010).

[b3] Kastner M. A. The single-electron transistor. Rev. Mod. Phys. 64, 849–858, 10.1103/RevModPhys.64.849 (1992).

[b4] Hanson R. & Awschalom D. D. Coherent manipulation of single spins in semiconductors. Nature 453, 1043–1049, 10.1038/nature07129 (2008). 18563155

[b5] Hanson R., Kouwenhoven L. P., Petta J. R., Tarucha S. & Vandersypen L. M. K. Spins in few-electron quantum dots. Rev. Mod. Phys. 79, 1217–1265, 10.1103/RevModPhys.79.1217 (2007).

[b6] Stopa M. Rectifying behavior in coulomb blockades: Charging rectifiers. Phys. Rev. Lett. 88, 146802, 10.1103/PhysRevLett.88.146802 (2002). 11955166

[b7] Scheibner R. . Quantum dot as thermal rectifier. New Journal of Physics 10, 083016, 10.1088/1367-2630/10/8/083016 (2008).

[b8] Zipper E., Kurpas M. & Maśka M. M. Wave function engineering in quantum dot—ring nanostructures. New J. Phys. 14, 093029, 10.1088/1367-2630/14/9/093029 (2012).

[b9] Somaschini C., Bietti S., Sanguinetti S., Koguchi N. & Fedorov A. Self-assembled gaas/algaas coupled quantum ring-disk structures by droplet epitaxy. Nanotechnology 21, 125601, 10.1088/0957-4484/21/12/125601 (2010). 20182013

[b10] Somaschini C., Bietti S., Koguchi N. & Sanguinetti S. Coupled quantum dot—ring structures by droplet epitaxy. Nanotechnology 22, 185602, 10.1088/0957-4484/22/18/185602 (2011). 21415467

[b11] Somaschini C., Bietti S., Koguchi N. & Sanguinetti S. Shape control via surface reconstruction kinetics of droplet epitaxy nanostructures. Applied Physics Letters 97, 203109, 10.1063/1.3511283 (2010).

[b12] Lauhon L. J., Gudiksen M. S., Wang D. & Lieber Ch. M. Epitaxial core-shell and core-multishell nanowire heterostructures. Nature 420, 57–61, 10.1038/nature01141 (2002). 12422212

[b13] Kurpas M., Kędzierska B., Janus-Zygmunt I., Maśka M. & Zipper E. Electrical control of spin relaxation time in complex quantum nanostructures. Acta Phys. Pol. 126, A20–A24 10.12693/APhysPolA.126.A-20 (2014).

[b14] Zhitenev N. B., Brodsky M., Ashoori R. C., Pfeiffer L. N. & West K. W. Localization-delocalization transition in quantum dots. Science 285, 715–718 10.1126/science.285.5428.715 (1999). 10426989

[b15] Szafran B., Peeters F. M. & Bednarek S. Electron spin and charge switching in a coupled quantum-dot21quantum ring system. Phys. Rev. B 70, 125310, 10.1103/PhysRevB.70.125310 (2004).

[b16] Janus-Zygmunt I., Kędzierska B., Gorczyca-Goraj A., Zipper E. & Maśka M. M. Quantum dot—ring nanostructure—a comparison of different approaches. Int. J. Mod. Phys. B 30, 1642013 10.1142/S0217979216420133 (2016).

[b17] Kurpas M. . Charge transport through a semiconductor quantum dot-ring nanostructure. J. Phys.: Condens. Matter 27, 265801, 10.1088/0953-8984/27/26/265801 (2015). 26052631

[b18] Barseghyan M., Manaselyan A., Laroze D. & Kirakosyan A. Impurity-modulated aharonov—bohm oscillations and intraband optical absorption in quantum dot—ring nanostructures. Physica E Low Dimens Syst Nanostruct. 81, 31–36 10.1016/j.physe.2016.02.012 (2016).

[b19] Zeng Z., Garoufalis C. S. & Baskoutas S. Linear and nonlinear optical susceptibilities in a laterally coupled quantum-dot—quantum-ring system. Phys. Lett. A 378, 2713–2718, 10.1016/j.physleta.2014.07.036 (2014).

[b20] Spałek J., Podsiadły R., Wójcik W. & Rycerz A. Optimization of single-particle basis for exactly soluble models of correlated electrons. Phys. Rev. B 61, 15676, 10.1103/PhysRevB.61.15676 (2000).

[b21] Kądzielawa A.. *H*_2_ and (*H*_2_)_2_ molecules with an ab initio optimization of wave functions in correlated state: electron—proton couplings and intermolecular microscopic parameters. New J. Phys. 16, 123022, 10.1088/1367-2630/16/12/123022 (2014).

[b22] Biborski A., Kądzielawa A. P. & Spałek J. Combined shared and distributed memory ab-initio computations of molecular-hydrogen systems in the correlated state: Process pool solution and two-level parallelism. Comp. Phys. Commun. 197, 7–16, 10.1016/j.cpc.2015.08.001 (2015).

[b23] For review see: Spałek J., Görlich E. M., Rycerz A. & Zahorbeński R.. The combined exact diagonalization—ab initio approach and its application to correlated electronic states and Mott—Hubbard localization in nanoscopic systems. J. Phys.: Condens. Matter 19, 255212, 10.1088/0953-8984/19/25/255212 (2007), pp. 1–43.

[b24] Fetter A. L. & Walecka J. D. Quantum Theory of Many-Particle Systems (Dover, 2003).

[b25] Hahn T. Cuba—a library for multidimensional numerical integration. Comp. Phys. Commun. 176, 712–713, 10.1016/j.cpc.2007.03.006 (2007).

[b26] Gnu Scientific Library (2015). URL http://www.gnu.org/software/gsl/, Date of access: 18/05/2016.

[b27] Biborski A. & Kądzielawa A. P. QMT: Quantum Metallization Tools Library (2014). URL https://bitbucket.org/azja/qmt, Date of access: 18/05/2016.

[b28] Szabo A. & Ostlund N. S. Quantum Chemistry: Introduction tp Advanced Electronic Structure Theory (McGraw-Hill Publishing Co., New York, 1989).

[b29] Kurpas M., Zipper E. & Maśka M. M. Physics of Quantum Rings, chap. Engineering of Electron States and Spin Relaxation in Quantum Rings and Quantum Dot-Ring Nanostructures, 455–479, 10.1007/978-3-642-39197-2_18 (Springer Berlin Heidelberg, Berlin, Heidelberg, 2014).

