# Supplementary material for: Dot-ring nanostructure: Rigorous analysis of many-electron effects
Source: Sci Rep. 2016 Jul 19;6:29887. doi: 10.1038/srep29887 (PMC4949483; doi:10.1038/srep29887)
Supplement: Supplementary Information [file srep29887-s1.pdf]

Supplementary Information

Dot-ring nanostructure: Rigorous analysis of many-electron effects

Andrzej Biborski[1], Andrzej P. Kądzielawa[2], Anna Gorczyca-Goraj[3], Elżbieta Zipper[3], Maciej M. Maśka[3], and Józef Spałek[1,2]

[1] Akademickie Centrum Materiałów i Nanotechnologii, AGH Akademia Górniczo-Hutnicza, Al. Mickiewicza 30, Kraków, Poland

[2] Instytut Fizyki im. Mariana Smoluchowskiego, Uniwersytet Jagielloński, ul. Łojasiewicza 11, PL-30-348 Kraków, Poland

[3] Instytut Fizyki, Uniwersytet Śląski, ul. Uniwersytecka 4, PL-40-007 Katowice, Poland

A Starting basis of single-particle wave-functions

The shapes of the ten selected single-particle real wave functions $\varphi_{nl}(\mathbf{r})$ forming a trial basis for the definition of the field operators in (1) are characterized below in Figs. S1 and S2. The method of calculating those wave-functions relies on solving the wave equation for a single electron for the potential energy depicted in Fig. 1. The details and the method accuracy is discussed elsewhere [8,17,29]. With the rotational symmetry of the potential in the DRN plane, those wave functions exhibit similarity to the hydrogenic-like functions for values of $l = 0, \pm 1, \pm 2$ respectively. This minimal basis with $M = 10$ components in Eq. (1) is sufficient to describe accurately the multiparticle states for $N_e = 2$ and 3 electrons analyzed in main text. A subsequent enlargement of the starting basis to $M = 18$ functions did not influence the accuracy of the presented results.

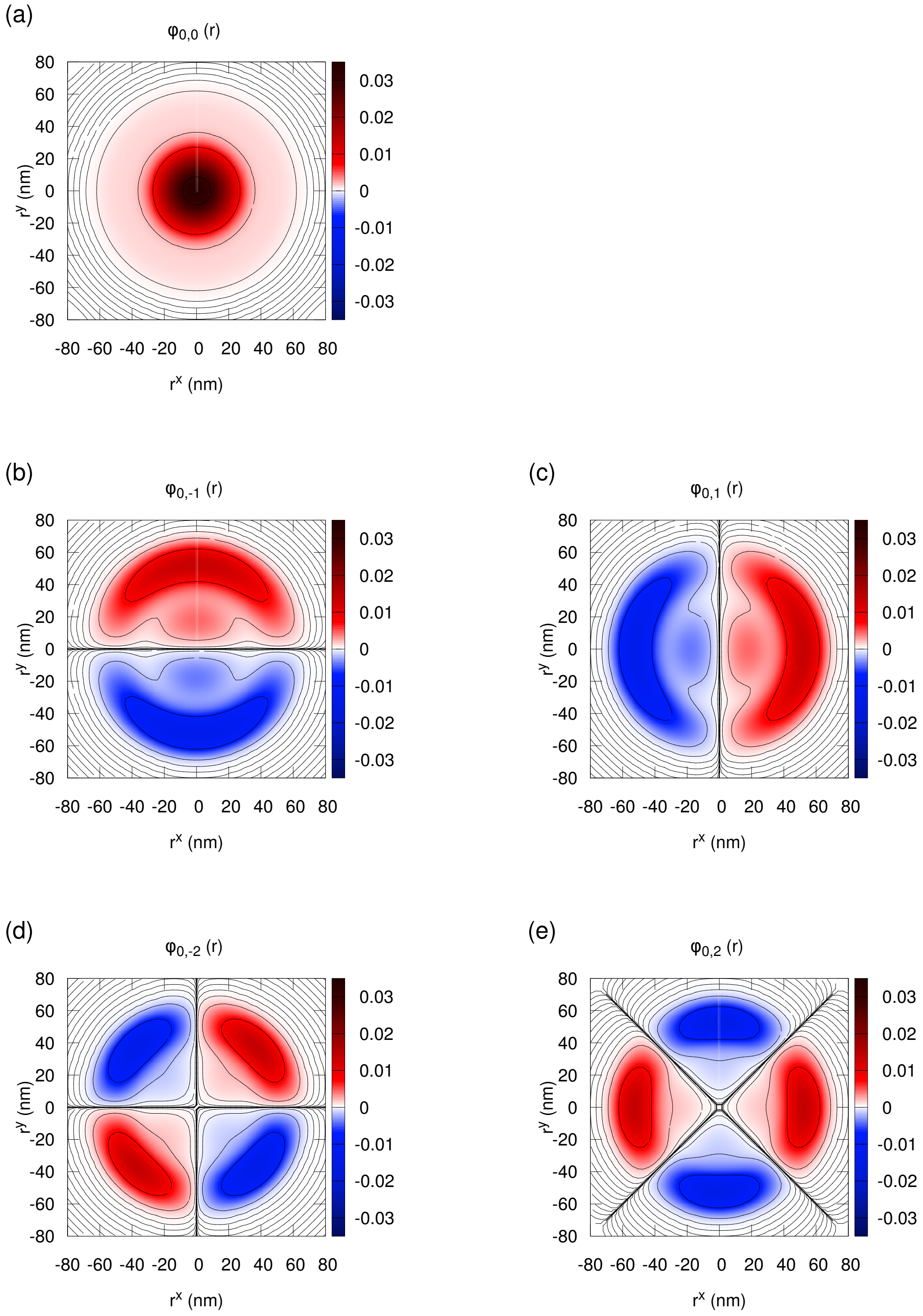

Figure S1. Starting single-particle wavefunctions for $n = 0$, $l = 0, \mp 1, \mp 2$ ((a) – (e)), respectively, all for the quantum dot potential $V_{\mathrm{QD}} = 0$, taken to define the field operator in Eq. (1). Note their similarity to the s, p_x, p_y, d_{xy} and $d_{x^2-y^2}$ atomic states, respectively.

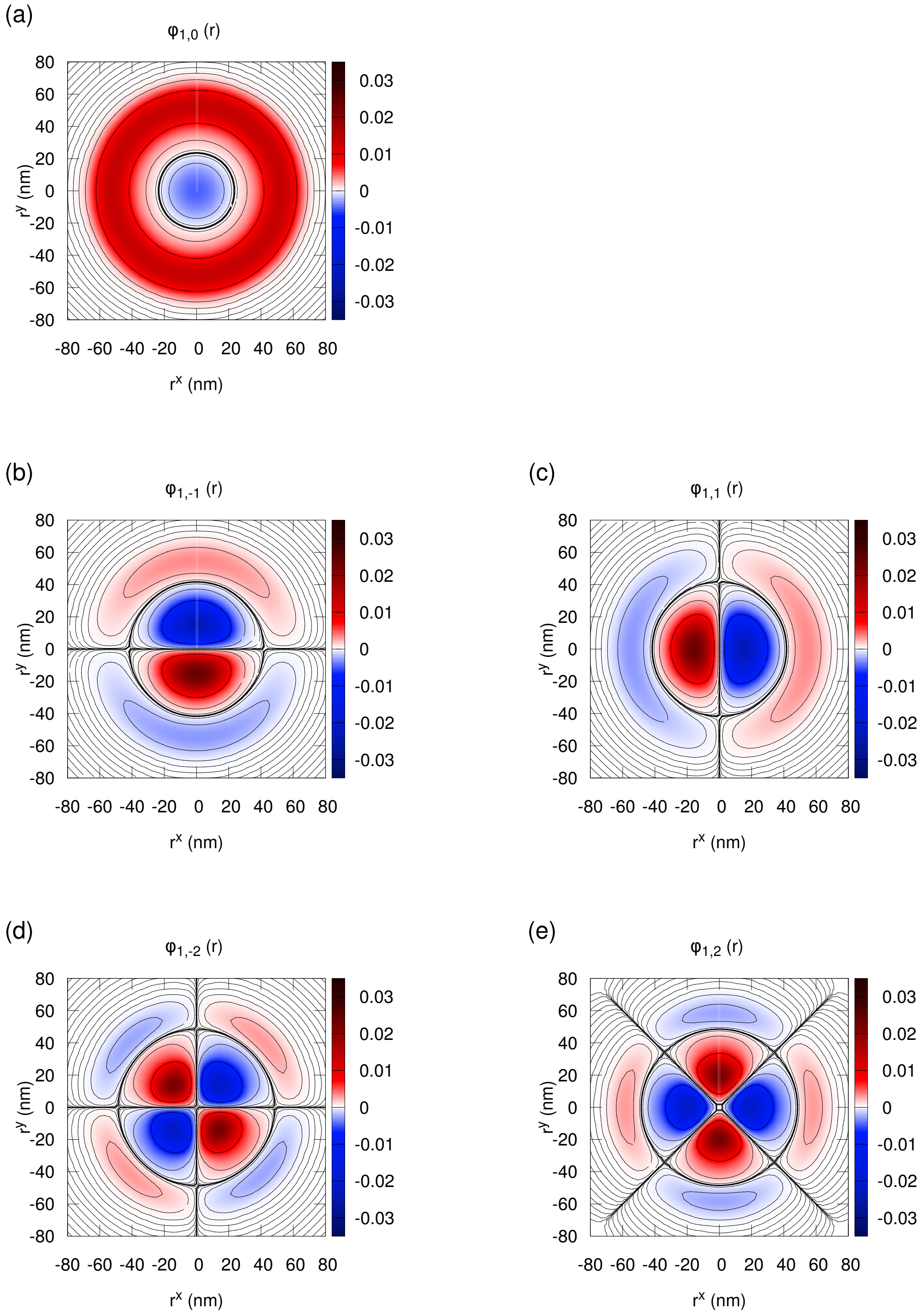

Figure S2. Starting single-particle wavefunctions for $n = 1$, $l = 0, \mp 1, \mp 2$ ((a) – (e)), respectively, all for the quantum dot potential $V_{\mathrm{QD}} = 0$, taken together with those depicted in Fig. S1 to define the field operator (1). The subsidiary (external) maxima and minima reflect the part associated with the presence of the ring.

B Detailed values of microscopic interaction parameters in Fock space

For the sake of completeness we provide the detailed numerical values of selected Coulomb interaction parameters as a function of the relative QD potential V_{QD}. Note that we have included also the 3- and 4-state interaction parameters, often ignored in many-particle considerations. Although the values of those last parameters are small, they are important as the corresponding number of such four-state terms (c.f. S5) is the largest and equal to 5040 (when we disregard symmetries leading to their degeneracy).

Table S1. All Hubbard intrastate repulsion amplitudes $U_i \equiv V_{iiii}$ (in meV) for different QD potentials.

V_{QD} (meV)	-6	-5	-4	-3	-2	-1	0	1	2	3	4	5	6
$U_{(0\ 0)}$	8.67	8.60	8.59	8.51	8.55	8.38	8.33	8.08	7.15	2.97	2.90	2.88	2.92
$U_{(0\ 1)}$	7.61	7.49	7.20	6.27	3.67	3.32	3.41	3.44	3.45	3.50	3.48	3.51	3.49
$U_{(0\ \bar{1})}$	7.63	7.47	7.14	6.21	3.63	3.29	3.39	3.47	3.43	3.49	3.49	3.51	3.53
$U_{(0\ 2)}$	3.19	3.23	3.28	3.29	3.28	3.29	3.28	3.32	3.28	3.30	3.27	3.27	3.26
$U_{(0\ \bar{2})}$	3.26	3.24	3.25	3.27	3.31	3.26	3.27	3.30	3.26	3.32	3.26	3.29	3.26
$U_{(1\ 0)}$	2.94	2.90	2.89	2.93	2.91	2.89	2.89	2.89	2.87	5.60	7.74	7.97	7.96
$U_{(1\ 1)}$	3.47	3.51	3.40	3.33	4.25	6.50	7.02	7.11	7.15	7.01	6.89	6.83	6.64
$U_{(1\ \bar{1})}$	3.46	3.46	3.42	3.28	4.27	6.50	6.97	7.12	7.08	7.02	6.88	6.83	6.66
$U_{(1\ 2)}$	6.18	6.31	6.27	6.26	6.02	5.93	5.86	5.76	5.61	5.35	5.18	4.74	4.36
$U_{(1\ \bar{2})}$	6.20	6.29	6.30	6.25	6.15	6.04	5.93	5.82	5.63	5.29	5.09	4.75	4.40

Table S2. Selected values of the interstate Coulomb repulsion amplitudes $K_{ij} \equiv V_{ijij}$ (in meV) for different QD potentials.

V_{QD} (meV)	-6	-5	-4	-3	-2	-1	0	1	2	3	4	5	6
$K_{(0\ 0),(0\ 1)}$	7.20	7.15	6.99	6.46	4.26	2.67	2.38	2.29	2.32	2.73	2.90	2.84	2.93
$K_{(0\ 0),(0\ 2)}$	2.38	2.28	2.25	2.26	2.23	2.22	2.23	2.22	2.30	2.74	2.88	2.91	2.91
$K_{(0\ 0),(1\ 0)}$	2.28	2.27	2.27	2.27	2.27	2.27	2.29	2.38	2.88	3.53	2.45	2.29	2.26
$K_{(0\ 0),(1\ 1)}$	2.28	2.31	2.43	2.90	5.03	6.57	6.81	6.72	6.37	3.47	2.44	2.34	2.31
$K_{(0\ 0),(1\ 2)}$	6.17	6.15	6.11	6.11	6.03	5.92	5.87	5.66	5.35	3.17	2.50	2.44	2.46
$K_{(0\ 1),(0\ \bar{1})}$	5.89	5.84	5.60	4.90	2.89	2.35	2.32	2.34	2.36	2.35	2.36	2.33	2.37
$K_{(0\ 2),(0\ \bar{2})}$	2.54	2.54	2.53	2.53	2.57	2.55	2.54	2.55	2.54	2.53	2.53	2.52	2.55
$K_{(1\ 1),(1\ \bar{1})}$	2.33	2.32	2.35	2.37	3.41	5.11	5.47	5.51	5.47	5.47	5.35	5.29	5.14

Table S3. Selected values of the interstate exchange integral $J_{ij} \equiv V_{ijji}$ (in meV) for different QD potentials.

V_{QD} (meV)	-6	-5	-4	-3	-2	-1	0	1	2	3	4	5	6
$J_{(0\ 0),(0\ 1)}$	2.40	2.37	2.31	2.03	1.00	0.24	0.10	0.08	0.20	1.20	1.52	1.53	1.55
$J_{(0\ 0),(0\ 2)}$	0.04	0.02	0.02	0.01	0.01	0.01	0.01	0.03	0.12	0.86	1.05	1.15	1.14
$J_{(0\ 0),(1\ 0)}$	0.04	0.03	0.03	0.03	0.04	0.05	0.08	0.17	0.62	1.28	0.25	0.09	0.05
$J_{(0\ 0),(1\ 1)}$	0.04	0.06	0.11	0.35	1.39	2.16	2.26	2.20	2.09	0.62	0.11	0.06	0.04
$J_{(0\ 0),(1\ 2)}$	1.09	1.06	1.06	1.06	1.04	0.98	0.97	0.94	0.88	0.30	0.11	0.10	0.10
$J_{(0\ 1),(0\ \bar{1})}$	0.88	0.87	0.76	0.66	0.38	0.48	0.53	0.55	0.57	0.56	0.57	0.58	0.56
$J_{(0\ 2),(0\ \bar{2})}$	0.34	0.36	0.37	0.36	0.39	0.37	0.35	0.40	0.36	0.38	0.38	0.39	0.39
$J_{(1\ 1),(1\ \bar{1})}$	0.57	0.57	0.53	0.47	0.43	0.71	0.80	0.79	0.80	0.79	0.77	0.77	0.73

Table S4. Selected correlated interstate hopping parameter values $C_{ij} \equiv V_{ijjj}$ (in meV) for different QD potentials.

V_{QD} (meV)	-6	-5	-4	-3	-2	-1	0	1	2	3	4	5	6
$C_{(0\ 0),(0\ 1)}$	0.05	-0.03	0.01	-0.06	-0.01	-0.01	0.01	0	0	-0.02	0	-0.01	0.03
$C_{(0\ 0),(0\ 2)}$	0	0.01	0	0	0	0	0	0	-0.01	0.02	0.02	0.01	0.01
$C_{(0\ 0),(1\ 0)}$	-0.38	-0.37	-0.37	-0.39	-0.44	-0.51	-0.65	-0.94	-1.65	-0.47	0.09	0.08	0.06
$C_{(0\ 0),(1\ 1)}$	0	-0.01	0	0	-0.05	0.02	-0.02	0	0.03	0	0	0	0
$C_{(0\ 0),(1\ 2)}$	0.03	0	-0.03	0.02	-0.03	-0.01	-0.01	0.02	0	-0.02	0	0.01	-0.01
$C_{(0\ 1),(0\ \bar{1})}$	0.03	0.01	0.01	-0.01	-0.01	-0.02	0	0	-0.01	0	-0.01	0	-0.01
$C_{(0\ 2),(0\ \bar{2})}$	-0.03	0.03	-0.01	0	0	0.02	-0.01	-0.01	0	0.03	-0.01	-0.02	-0.01
$C_{(1\ 1),(1\ \bar{1})}$	0	0.02	0	0	0.01	-0.03	-0.02	0.04	0	0.02	-0.02	0	-0.01

Table S5. Selected three- and four-state parameters $V_{[ijkl]}$ (in meV) for different QD potentials.

V_{QD} (meV)	-6	-5	-4	-3	-2	-1	0	1	2	3	4	5	6
$V_{(0\ 0),(0\ 1),(0\ 1),(0\ 2)}$	0.34	0.27	0.23	0.23	0.21	0.15	0.14	0.19	0.37	0.97	1.08	1.10	1.12
$V_{(0\ 0),(0\ 1),(0\ \bar{2}),(0\ \bar{1})}$	0.19	0.14	0.11	0.08	0.06	0.06	0.07	0.12	0.25	0.68	0.80	0.81	0.82
$V_{(0\ 0),(0\ \bar{1}),(0\ 1),(0\ \bar{2})}$	0.35	0.27	0.23	0.23	0.21	0.15	0.15	0.19	0.37	0.95	1.06	1.11	1.09
$V_{(0\ 0),(0\ 2),(0\ 2),(0\ \bar{1})}$	0	0	0	0	0	0	0	0	0	0.03	0.04	-0.01	0.01
$V_{(0\ 1),(0\ 1),(1\ 0),(0\ 0)}$	0.04	0.03	0.04	0.09	0.18	0.18	0.20	0.26	0.48	0.67	0.29	0.18	0.12
$V_{(1\ 0),(0\ 2),(1\ \bar{1}),(0\ \bar{1})}$	-0.16	-0.19	-0.24	-0.40	-0.57	-0.33	-0.23	-0.19	-0.19	-0.28	-0.27	-0.27	-0.28

C DRN in the correlated state: 2- and 3-electrons particle density

Here we provide the characteristics of the first excited state for $N_e = 2$ for two additional values of V_{QD}.

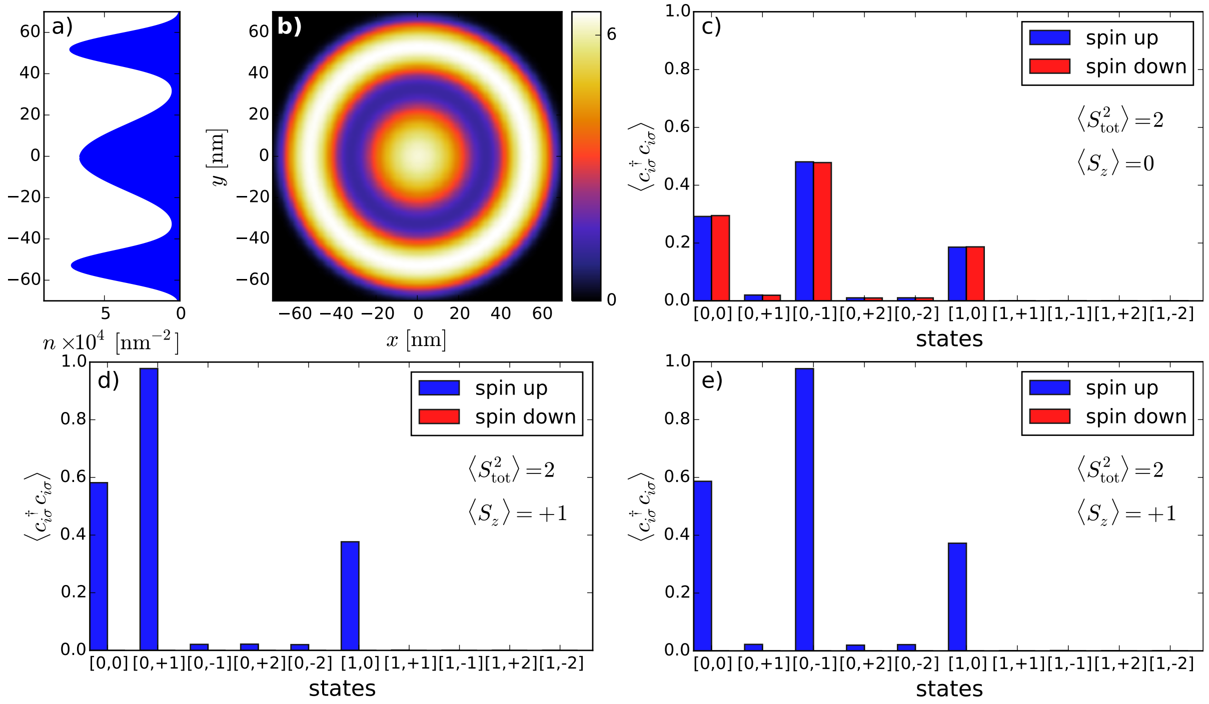

Figure S3. The same as in Fig. 5, but for $V_{\text{QD}} = 2$ meV.

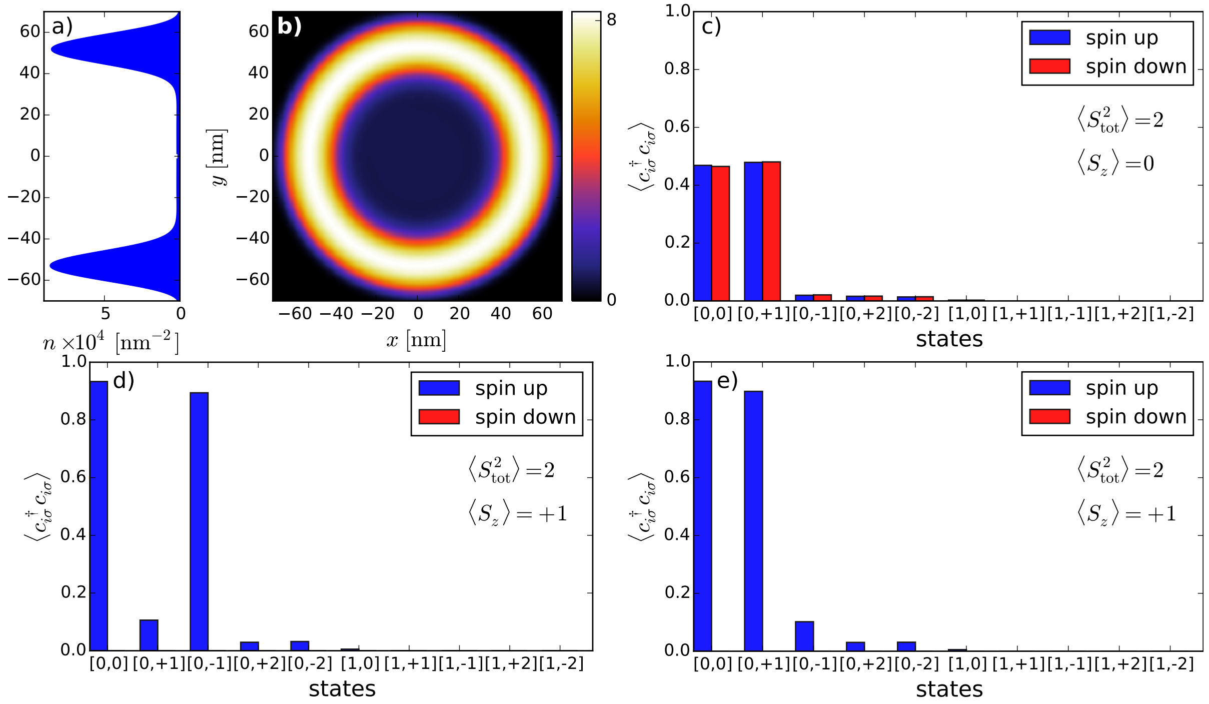

Figure S4. The same as in Fig. 5, but for $V_{\text{QD}} = 4$ meV.

D The degree of degeneracy in the ground- and first-excited state

In this Supplement we list the degeneracies of the multiparticle states. The first factor of the total degeneracy is due to the S^z_{tot} ($2S^z_{tot}+1$ values). The additional degeneracy represents an emergent chirality and is related to the number of ways the single-electron current can compose the total orbital current.

Table S6. The degrees of degeneracy for different QD potentials, with $N_e = 2, 3$.

	2 electrons				3 electrons			
	ground state		first-excited state		ground state		first-excited state	
V_{QD} (meV)	deg.	S_{tot}	deg.	S_{tot}	deg.	S_{tot}	deg.	S_{tot}
-6	1	0	3×2	1	2×3	1/2	2×2	1/2
-5	1	0	3×2	1	2×3	1/2	2×2	1/2
-4	1	0	3	1	2×3	1/2	4×2	3/2
-3	1	0	3	1	2×3	1/2	4×2	3/2
-2	1	0	3	1	2×3	1/2	4×2	3/2
-1	1	0	3	1	2×3	1/2	4×2	3/2
0	1	0	3	1	2×3	1/2	4×2	3/2
1	1	0	3	1	2×3	1/2	4×2	3/2
2	1	0	3×2	1	4	3/2	2×2	1/2
3	1	0	3×2	1	4	3/2	2×2	1/2
4	1	0	3×2	1	4	3/2	2×2	1/2
5	1	0	3×2	1	4	3/2	2×2	1/2
6	1	0	3×2	1	4	3/2	2×2	1/2

E Average total momentum along the z-axis

We define the average total momentum along the z-axis as

$$\langle L^z\rangle \equiv \sum_\sigma \left\langle \Phi \middle| \int d^3r \hat{\Psi}^\dagger_\sigma(\mathbf{r}) \hat{L}^z \hat{\Psi}_\sigma(\mathbf{r}) \middle| \Phi \right\rangle = \sum_{\sigma,i,j} \int d^3r \varphi^*_{i\sigma} \hat{L}^z \varphi_{j\sigma} \left\langle \hat{c}^\dagger_{i\sigma} \hat{c}_{j\sigma} \right\rangle, \tag{S1}$$

where $|\Phi\rangle$ is the state in the Fock space, $\hat{\Psi}_\sigma(\mathbf{r})$ is the field operator (1), $\{\varphi_i\}$ is the single-particle wave-function basis, i and j are the pairs of quantum numbers $[n,l]$ defining the single-particle state, and

$$\hat{L}^z \equiv -i\hbar\partial_\phi \tag{S2}$$

is the single-particle z-component momentum operator. To calculate (S1) we must calculate how (S2) influences the single-particle wave-function basis $\{\varphi_i\}$. First, let us consider (S2) acting on the simpler single-particle wave-function basis $\{\psi_i\}$

$$\hat{L}^z \psi_{nl}(\mathbf{r}) \equiv -i\hbar\partial_\phi \psi_{nl}(\mathbf{r}) = -i\hbar\partial_\phi R_{nl}(r) e^{il\phi} = \hbar l \psi_{nl}(\mathbf{r}). \tag{S3}$$

Now, $\hat{L}^z$ acting $\{\varphi_i\}$ comes down to three cases

$$l=0: \quad -i\hbar\partial_\phi \varphi_{n0}(\mathbf{r}) = -i\hbar\partial_\phi \psi_{n0}(\mathbf{r}) = 0, \tag{S4a}$$

$$l>0: \quad -i\hbar\partial_\phi \varphi_{nl}(\mathbf{r}) = -i\hbar\partial_\phi \frac{\psi_{nl}(\mathbf{r}) + \psi_{n\bar{l}}(\mathbf{r})}{\sqrt{2}} = i\hbar l \frac{\psi_{nl}(\mathbf{r}) - \psi_{n\bar{l}}(\mathbf{r})}{\sqrt{2}i} = i\hbar l \varphi_{n\bar{l}}(\mathbf{r}), \tag{S4b}$$

$$l<0: \quad -i\hbar\partial_\phi \varphi_{nl}(\mathbf{r}) = -i\hbar\partial_\phi \frac{\psi_{n\bar{l}}(\mathbf{r}) - \psi_{nl}(\mathbf{r})}{\sqrt{2}i} = i\hbar l \frac{\psi_{n\bar{l}}(\mathbf{r}) + \psi_{nl}(\mathbf{r})}{\sqrt{2}} = i\hbar l \varphi_{n\bar{l}}(\mathbf{r}). \tag{S4c}$$

Eventually, due to the orthogonality of basis $\{\varphi_i\}$ the only non-zero elements in the sum (S1) are

$$\langle L^z\rangle = \sum_{\sigma,n,l>0} i\hbar l \left(\left\langle \hat{c}^\dagger_{n\bar{l}} \hat{c}_{nl} \right\rangle - \left\langle \hat{c}^\dagger_{nl} \hat{c}_{n\bar{l}} \right\rangle \right). \tag{S5}$$

It means that as long as the symmetry condition $\left\langle \hat{c}^\dagger_{n\bar{l}} \hat{c}_{nl} \right\rangle = \left\langle \hat{c}^\dagger_{nl} \hat{c}_{n\bar{l}} \right\rangle$ is fulfilled (which is the case here), the average total momentum along z-axis will be equal to zero.
